# Automating the Shared Resource Laboratory Using Computer Scripts: A Case Report

**DOI:** 10.1002/cyto.a.23775

**Published:** 2019-04-29

**Authors:** Christopher Hall, Laura Brown, Jennifer Graham, Sam Thompson, Bee Ling Ng

**Affiliations:** ^1^ Cytometry Core Facility Wellcome Sanger Institute, Genome Campus Hinxton, CB10 1SA UK; ^2^ Finance Division, University of Cambridge Cambridge, CB2 1TT UK

**Keywords:** Shared Resource Laboratory, SRL, flow cytometry, automation, scripting, facility management, XDP, Influx, Python, R

## Abstract

Shared resource laboratories (SRLs) offer instrumentation, training, and support to investigators and play an important role in the progress and development of science. To facilitate daily tasks and to provide an effective service, we have made use of computer scripts; a list of computer commands that are processed sequentially, to automate tasks in our flow cytometry facility. Using Python and an application programming interface (API), we automate user communication and produce a daily schedule display screen. We exploit the accessible nature of open standards to use R and Python to analyze and backup data from the BD Influx cell sorter. Finally, we show that through simple scripting, we can add value to an existing service by producing sort statistics from the Beckman Coulter XDP cell sorter. With these five examples, we demonstrate and wish to inspire other SRLs that the use of scripts helps to improve work efficiency, can solve problems, and can enhance the service provided by the SRL. © 2019 The Authors. Cytometry Part A published by Wiley Periodicals, Inc. on behalf of International Society for Advancement of Cytometry.

The use of computers in society and science is ubiquitous. In a shared resource laboratory (SRL) they can be found, among other places, in the offices, controlling the instrumentation, as the servers hosting the booking calendars, and storing scientific data. One beneficial use of computers is to automate repetitive tasks. This is done in one of two ways; using a purpose‐built solution, such as accounting software that can calculate costs and generate bills, or in an ad hoc manner using a scripting language to tailor a solution to a specific need. A computer script is a list of commands that are executed in a set order, similar to a “to do” list, which can be repeated at specific times or on request to produce a predictable result. Common scripting languages include Python (general purpose), PowerShell (Microsoft Windows automation), Bash (UNIX automation), and R (statistics). Scripting is used extensively in many industries to automate routine work such as updating websites with PERL 1, controlling machinery with LabView 2, and trading shares with Python 3.

The emergence of the SRL model has brought about many benefits including reduced costs resulting from increased negotiation leverage, the concentration of expertise allowing better and more innovative running of instrumentation, and reduced bureaucratic burden with one management structure operating many instruments 4. To best realize these benefits, it would be sensible to identify repetitive tasks or issues and to automate as many as possible.

In this SRL communication, we will highlight examples where we have automated a task using computer scripts, how these can be applied to other SRLs and where we see future opportunities. These tasks are grouped into three sections; solving SRL management issues, saving time, and extending the capabilities of instrumentation and services. Through this case study, we show that time invested in the short term developing these scripts can lead to long‐term SRL improvements and hope to inspire other SRLs to adapt these examples, or to create new scripts, to achieve similar benefits.

## Availability

All the scripts mentioned in this SRL communication paper are freely available on the code development and sharing website Github at www.github.com/sangercytometry. The names of the scripts are found in brackets in the titles of each subheading. It is important to emphasize that all the scripts described in this article were written and deployed by the SRL without the help of a professional programmer or IT support. This is possible because of the collaborative nature of computer programming where prebuilt packages are freely made available to perform set tasks and that there are very active online communities of people willing to ask and to answer questions. All the scripts are extensively annotated to allow easy modification and to make them easier for others to understand.

## Case Studies

### Solving Problems

The bespoke nature of a script allows the SRL to tackle challenges either unique to a specific facility or problems too small to be commercially viable for others to solve. Here, we present two scripts we have written to solve the issues that we have experienced in our SRL, but will also be beneficial to other SRLs.

#### Solving the issue of instruments left on unintentionally (PPMSemail)

**Figure 1:**
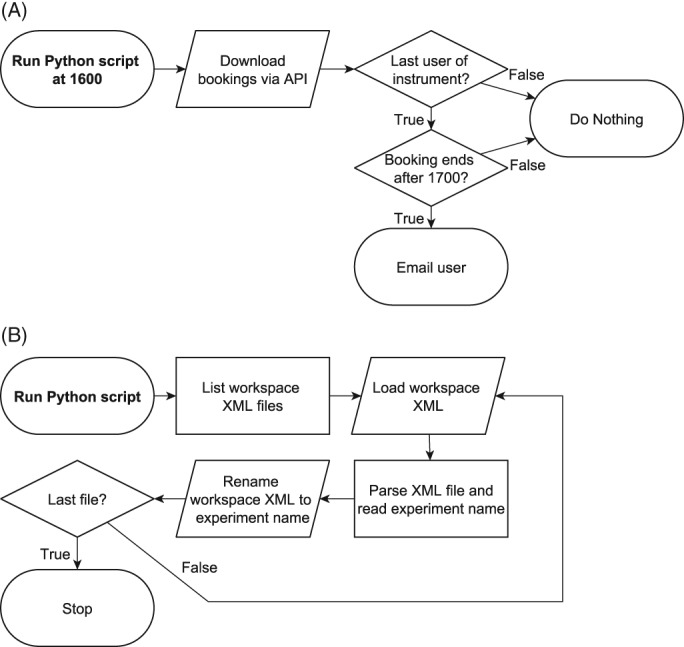
Process flowchart of (**A**) the script that automatically emails the last user if their session ends after 5 pm (when our SRL staff leave) and (**B**) the script that renames each BD influx workspace from its generic name to a verbose experiment name.

A common problem in our SRL is users forgetting to switch off instruments and leaving them on overnight. This results in decreased laser life, increased energy usage, and the possibility of instrument damage. To tackle this problem, we have developed a script using Python to contact our users prior to their session. The PPMSemail script uses the application program interface (API) of our booking calendar software, PPMS (Stratocore, France). An API is a communication protocol that allows access to an instrument or service without needing to understand its underlying architecture. The API is designed by the manufacturer who produces a set list of commands in which the end user can use. The PPMSemail script runs at a scheduled time during the day, identifies the last user of each machine, and where the session finishes outside office hours, sends an email to the user informing them or their responsibility to shut down the instrument. See Figure 1A for a process overview.

In 2016, the last full year before the script was deployed, we had 11 documented incidents of instruments being left on overnight; in 2018, it was reduced to two incidents, a reduction of just over 80%.

#### Solving the issue of vague Becton Dickson (BD) Influx workspace names (Influx‐workspace‐renamer)

The BD Influx cell sorter (Becton Dickinson, NJ) stores sort templates and saved workspaces (a previously saved sort) as XML documents. XML is an internationally standardized document format that is both human and machine readable 5. When opening a workspace, the BD Influx software, named SortWare, reads each XML file and displays pertinent information such as the date created and experiment name. These XML files are saved onto the PC with sequentially numbered filenames starting with “configuration_1.xml.” This setup presents two issues for data management; first, that as the number of saved workspaces grows SortWare takes progressively longer to load the workspace details; second, that when backing up the instrument data the operator cannot identify which saved workspace belongs to which experiment. To solve these issues, we developed a Python script that parses the XML data and renames the files to match the details that are displayed in SortWare, that is, the experiment name from the “name” tag in the XML file. Running the Influx‐workspace‐renamer script results in a list of XML files with verbose file names, see Figure 1B for the process flowchart. The renamed files can now be stored away from SortWare resulting in reduced load times and can be easily recognized by the operator when they need to be recovered. Serendipitously, we discovered that SortWare does not require that the XML files have a set name to successfully open a workspace so we can apply this script, with the new descriptive filenames, to the live installation if desired.

### Saving Time

Of interest to SRLs automating processes is the amount of time saved performing a task. We employ a number of time saving scripts, including batch booking of sessions, counting and sorting files, and exporting usage data. Here, we describe one script that we use to save time when exporting user data.

#### Exporting index data from the BD Influx flow cytometer (R‐Scripts/Influx2csv v1.32.R)

The standardized nature of flow cytometry data files (FCS) allows them to be analyzed by any application that can read the format 6. This has led to the proliferation of data analysis programs, allowed researchers to produce innovative data analysis methodologies, such as FlowSOM 7, and allowed them to bring analysis concepts from other fields into flow cytometry, such as viSNE 8. We have made use of R to enhance how we manage our user sort data that contains index information. R was used in order to take advantage of prewritten packages (a collection of R functions written by the R community) that are specific to flow cytometry and data manipulation.

**Figure 2:**
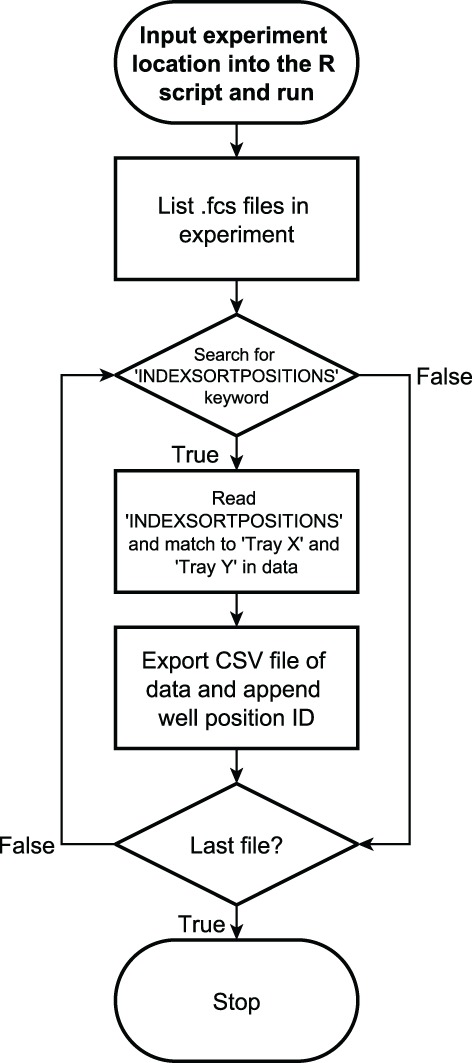
Process flowchart showing the script that exports the BD influx index sort statistics from the generated FCS file using R.

The BD Influx cell sorter stores index data (sort position of deposited cell) in the FCS file as a keyword and as three additional columns in the data stream. The keyword, “INDEXSORTPOSITIONS,” is a dictionary of the exact position of the stepper motor associated with a well position ID. The three columns in the data stream contain the stepper motor position in X and Y coordinates (“Tray X”, “Tray Y”) and a value labeled “Sort.Result.Bits” to indicate whether the event was sorted. We have written a script that takes this information and produces a CSV file for use by the user for data analysis. This script uses an R package called flowCore 9 to read the FCS file, reformats the data into a R data frame (data table), and compares the Tray X and Tray Y positions with the coordinates stored in the INDEXSORTPOSITIONS keyword. To do this, we have taken advantage of a data preparation package called fuzzyjoin 10, which allows us to associate the XY coordinates to the keyword coordinates despite the fact they do not match exactly. We then append the well positions to the FCS data and export it as a CSV table. A process flowchart for this script can be found in Figure 2.

There are three advantages to this method over using the inbuilt index data export function in the BD Influx software. First, that it is faster to export the data using this script because it can perform batch exports, which is not possible in the BD software (v1.2.0.142). Second, there is more control over how the data are processed; which transformation is applied, if any, and can be directly integrated into an analysis workflow. Finally, the data processing is transparent for reviewers, they can see exactly how the data are analyzed at every point in a workflow.

### Pushing Boundaries

By writing scripts, it is possible to go beyond the original design of the instrument or software. Here, we show two examples, one to overcome a limitation of a cell sorter and the other to extend the capabilities of an existing service.

#### Enabling the Beckman Coulter XDP to save sort statistics (XDP‐sort‐statistics)

**Figure 3:**
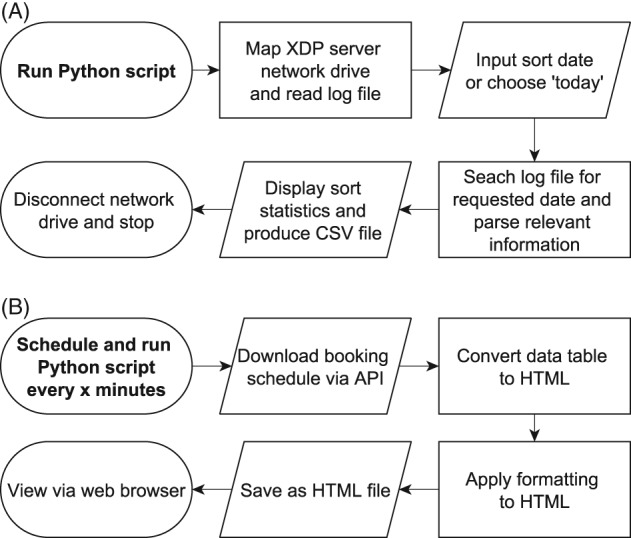
Process flowchart showing (A) the script that parses the XDP server log file into a sort statistics CSV file, and (**B**) the script that downloads and reformats our daily booking schedule for display via a web browser.

The Beckman Coulter MoFlo XDP (Beckman Coulter, IN) is a cell sorter and as part of the usual sort workflow the operator will record the number of cells sorted and the sort efficiency. To record these data, the operator either has to make a screenshot of the information and save it as an image, has to copy it to a spreadsheet, or has to write it onto a piece of paper. This is not an ideal situation as transcribing information can introduce errors, using images or hand written numbers introduces an extra layer of complexity, and there is no fall back in the case of an error. The BD Influx cell sorter records this information as a PDF document and the Sony SH800 cell sorter (Sony Biotechnology, Japan) stores it in an experiment database. We discovered that the computer server which lies between the MoFlo XDP instrument electronics and the controlling desktop PC stores the sort information in the form of a log file. Using a script written in Python we copied the log file, parsed the information, and produced a spreadsheet that can be given to the user. Figure 3A provides a process overview of the XDP‐sort‐statistics script.

#### Producing a display screen of the booking schedule (PPMS‐to‐HTML‐table)

We use the commercial scheduling software PPMS (Stratocore, France) that allows us to interact with it using an API. By using this capability, we have extended the utility of the software by producing a large laboratory display that shows, in real time, the booking schedule. We did this to improve our laboratory management by allowing the easy viewing of each instruments schedule and to reduce disputes about overrunning sessions. First, we employ the Requests package 11 to download a copy of the current day's booking from our scheduling software. To understand what to request from the scheduling software, we used the API help documentation from our booking calendar vendor. Next, we use the data manipulation package petl 12 to extract the relevant information from the downloaded file. To produce the HTML (web format) table from the downloaded schedule we use code from the code sharing website rosettacode.org. This website provides code examples in many languages of common programming tasks and we use the code called “CSV_to_HTML_translation#Python.” The final section of code colorized the table to provide increased visual clarity. This is all done automatically, via a scheduled task, every 10 min on a small form factor PC attached to a TV and the resulting HTML file is displayed through a web browser. The process overview can be found in Figure 3B.

## Discussion

We have shown that through the application of computer scripts to situations often encountered by the SRL we can save time, effort, avoid mistakes, fix problems, and extend capabilities. This is common practice in other industries, particularly computer science, and we have found that by applying it to our facility we have reduced the management burden and increased our productivity.

The programming languages we used, Python and R, are relatively easy to learn and well documented. They can be applied to any problem found in the SRL that involves a computer and is repetitive or involves responding to an event. Table 1 shows the amount of time the SRL spends on each repetitive task in 1 year, rounded to the nearest minute, hour, day, or month. We would suggest any task that is repeated often is worth trying to automate, as highlighted in gray. One major benefit not discussed in detail in this article is that through automation, the SRL cannot forget to do a task. If there is an important task that must be done every week then having the security of a scheduled script could be very beneficial.

**Table 1:** The amount of time taken by repeated tasks more than 1 year rounded to the nearest whole value

	FREQUENCY OF TASK (TOTAL PER YEAR)			
TASK DURATION	MONTHLY (12)	WEEKLY (52)	DAILY (365)	10 × DAY (3,650)
1 s	1 min	1 min	6 min	1 h
10 s	2 min	9 min	1 h	10 h
1 min	12 min	1 h	6 h	3 days
2 min	24 min	2 h	12 h	5 days
10 min	2 h	9 h	3 days	1 month
1 h	1 day	2 days	15 days	5 months
2 h	1 day	4 days	1 month	
1 day	12 days	52 days		

The cells highlighted in grey show the area where automation would be beneficial in saving time. It also indicates how long should be allocated to automating the task if the sole purpose is saving time over a year.

Providing ongoing support for the scripts is important as the demands of the SRL will change over time, for example, when instrument names are changed the scripts need altering to reflect this. A computer with 100% uptime is required to maintain the automation and it must be monitored as when it fails, the automation will fail with it. Table 1 indicates how much time can be saved through task automation; obversely, it can also be used to indicate how much time should be spent in creating the automation if the only benefit is saving time. It is difficult to estimate how long it took to create each of the scripts mentioned in this article because they were written in free time between sorting in a piecemeal fashion. What can be said is that each subsequent piece of coding took less effort to complete because the author gained experience and had built up a personal library of code examples. A central tenet of programming is not to rewrite code to do the same job again. This manifests itself in computer programs as reusable blocks of code (also known as functions), which can be called to do a job, rather than writing the code again. This principle also applies across different programs as many of the functions can be reused. For example, our PPMS Batch scripts (PPMSBatchScripts) were written in minutes because they reused code already written for the display screen script mentioned earlier (PPMS‐to‐HTML‐table) and many of the R flow cytometry data analysis scripts reuse the same data import, transformation, and visualization code, with added specification depending upon the data to be analyzed.

We have employed many more scripts then the ones mentioned in this SRL communication, many of which can be found at www.github.com/sangercytometry, but we also foresee future opportunities in automated instrument backup, centralizing QC data, and remotely monitoring sample quality. The bespoke nature of these scripts means that they can be applied to most problems that an SRL will encounter. This is only possible thanks to the open standards of the FCS, XML, and HTML file formats. It would be beneficial to the cytometry community if the instrument vendors and service providers were to continue to use these standards; such as BD using XML to save experiment settings, and to provide ways to interface with their products without risk of damaging them and through APIs like those provided by Stratocore in their booking calendar software. A prime example of where these capabilities have been used to the cytometry communities advantage is in the development of the “slice and dice” sort methodology where the BD Influx was manipulated outside the manufacturer's software to perform sorting based upon population diversity rather than population distribution 13. By continuing to embrace open standards the future of the cytometry community, and science, will be far richer.
